# A non-volatile cryogenic random-access memory based on the quantum anomalous Hall effect

**DOI:** 10.1038/s41598-021-87056-7

**Published:** 2021-04-12

**Authors:** Shamiul Alam, Md Shafayat Hossain, Ahmedullah Aziz

**Affiliations:** 1 Department of Electrical Engineering and Computer Science, University of Tennessee, Knoxville, TN 37996 USA; 2 Department of Electrical Engineering, Princeton University, Princeton, NJ 08544 USA

**Keywords:** Electrical and electronic engineering, Electronic devices, Electronic properties and devices

## Abstract

The interplay between ferromagnetism and topological properties of electronic band structures leads to a precise quantization of Hall resistance without any external magnetic field. This so-called quantum anomalous Hall effect (QAHE) is born out of topological correlations, and is oblivious of low-sample quality. It was envisioned to lead towards dissipation-less and topologically protected electronics. However, no clear framework of how to design such an electronic device out of it exists. Here we construct an ultra-low power, non-volatile, cryogenic memory architecture leveraging the QAHE phenomenon. Our design promises orders of magnitude lower cell area compared with the state-of-the-art cryogenic memory technologies. We harness the fundamentally quantized Hall resistance levels in moiré graphene heterostructures to store non-volatile binary bits (1, 0). We perform the memory write operation through controlled hysteretic switching between the quantized Hall states, using nano-ampere level currents with opposite polarities. The non-destructive read operation is performed by sensing the polarity of the transverse Hall voltage using a separate pair of terminals. We custom design the memory architecture with a novel sensing mechanism to avoid accidental data corruption, ensure highest memory density and minimize array leakage power. Our design provides a pathway towards realizing topologically protected memory devices.

## Introduction

Electronic bands of non-trivial topology give rise to quantum phenomena at macroscopic scale. Under a strong magnetic field, two-dimensional electron systems exhibit such a quantum state- the quantum Hall effect, i.e. the quantization of Hall resistance^1^. Back in 1988, Haldane^2^ predicted that a quantum Hall state can arise even in the absence of an external magnetic field. This new state became known as the quantum anomalous Hall effect (QAHE). However, it took more than two decades for the first experimental realization of QAHE, when it was demonstrated in a magnetic topological insulator [Cr-doped Bi(Sb)_2_Te_3_]^3–10^. The experimental fingerprint of QAHE is straightforward: The Hall resistance is quantized to h/*v*e^2^ (where $\nu ,$ the so-called Chern number, is an integer that depends on the topological properties of the band structure) at zero magnetic field. Such simple and universal values stem from the current being carried by lossless edge channels which require neither high electron mobility^11,12^ nor external magnetic fields. Moreover, on a given edge, the current flows only in one direction. Thanks to such transport properties which are also immune to sample complexities^11,12^, QAHE can potentially be useful in spin-filtering^11^, resistance metrology^13^ and topological quantum computing^14^. However, how we can materialize these prospects and come up with a QAHE-based electronic device have remained elusive so far.

Here we bridge the gap between the QAHE physics and device architecture to build the framework of a scalable non-volatile memory. We utilize the quantization of Hall resistance in QAHE to design a memory cell and then construct a 3D cross-point memory array capable of efficient read and write operations.

## Design of a QAHE based memory

To design our memory cell, we use the intrinsic QAHE reported in a twisted bilayer graphene (tBLG) on hexagonal boron nitride (hBN) moiré heterostructure, where the quantization of Hall resistance persists at temperatures as high as 6 K^1^. This is significantly higher compared to the magnetic topological materials, such as transition metal doped (Bi, Sb)_2_Te_3_ thin films^15–18^ and MnBi_2_Te_4_^19^.With the ongoing thrust in material discoveries, room-temperature applications may become feasible. Very recent works on QAHE in TbMn_6_Sn_6_, MnBi_2_Te_4_ offer such prospects^20–22^.

**Figure 1:**
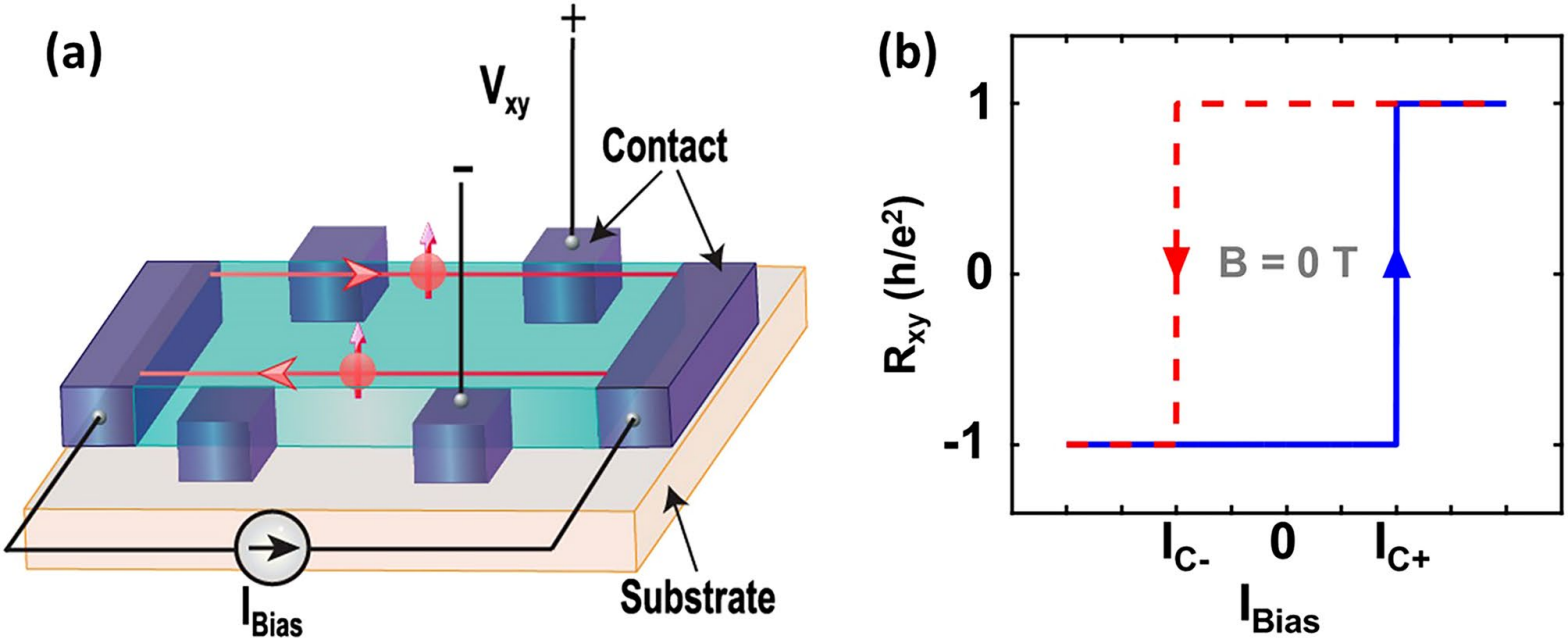
Schematic representation of quantum anomalous Hall effect. (**a**) Schematic of our device, where the red circles represent the electron and the red arrows show the spin of the electrons. A gate voltage can be applied at the back of the substrate to control the electronic density. *V*_*xx*_ and *V*_*xy*_ are the longitudinal and transverse (Hall) voltages, respectively, developed in response to the bias current, *I*_Bias._ (**b**) Illustration of the Hall resistance *R*_*xy*_ versus the bias current, *I*_Bias_ at zero external magnetic field. *I*_*C−*_ and *I*_*C*+_ are two critical values of *I*_Bias_ which determine the hysteretic switching of *R*_*xy*_ between *− h/e*^*2*^ and *h/*e^2^.

We start by delineating the dynamics of a QAH insulator and how it can be used as a non-volatile memory cell. Figure 1a illustrates the schematic of a QAH insulator with electric contacts to apply bias current and to measure both longitudinal and transverse (Hall) voltages. The QAH states occur when an applied gate voltage tunes the bulk carrier density to close to zero. Then, the Hall resistance shows a hysteretic switching with the change of magnetic field. Also, magnetic domains of the ferromagnetic materials (for example, tBLG) strongly interact with the applied electrical current, which provides the electrical control over the polarization of the magnetic domains^23^. It has also been shown^1^ that the switching of Hall resistance driven by DC bias current is similar to that driven by magnetic field. We leverage this tunability in our design. Broadly, our design can be implemented with any material platform harboring an electrically tunable QAHE. Note that the exact reason behind the electrical switching of QAHE in tBLG awaits a comprehensive understanding. Orientation of the graphene layers as well as the interaction between the electrical current and ferromagnetic domains in tBLG may play a role^22^. It is also worthwhile to add that electrically tunable QAHE is not unique in tBLG; tri-layer graphene (twisted monolayer-bilayer graphene) also shows similar tunability^24,25^.

Figure 1b shows a schematic of the hysteretic switching of Hall resistance *R*_*xy*_ (= *V*_*xy*_*/I*_Bias_) with the bias current at absolutely zero external magnetic field. Here, we mark two critical values of bias current, *I*_*C-*_ and *I*_*C*+_, which denote the values of bias current required for the switching of Hall resistance between ± *h/e*^2^.

**Figure 2:**
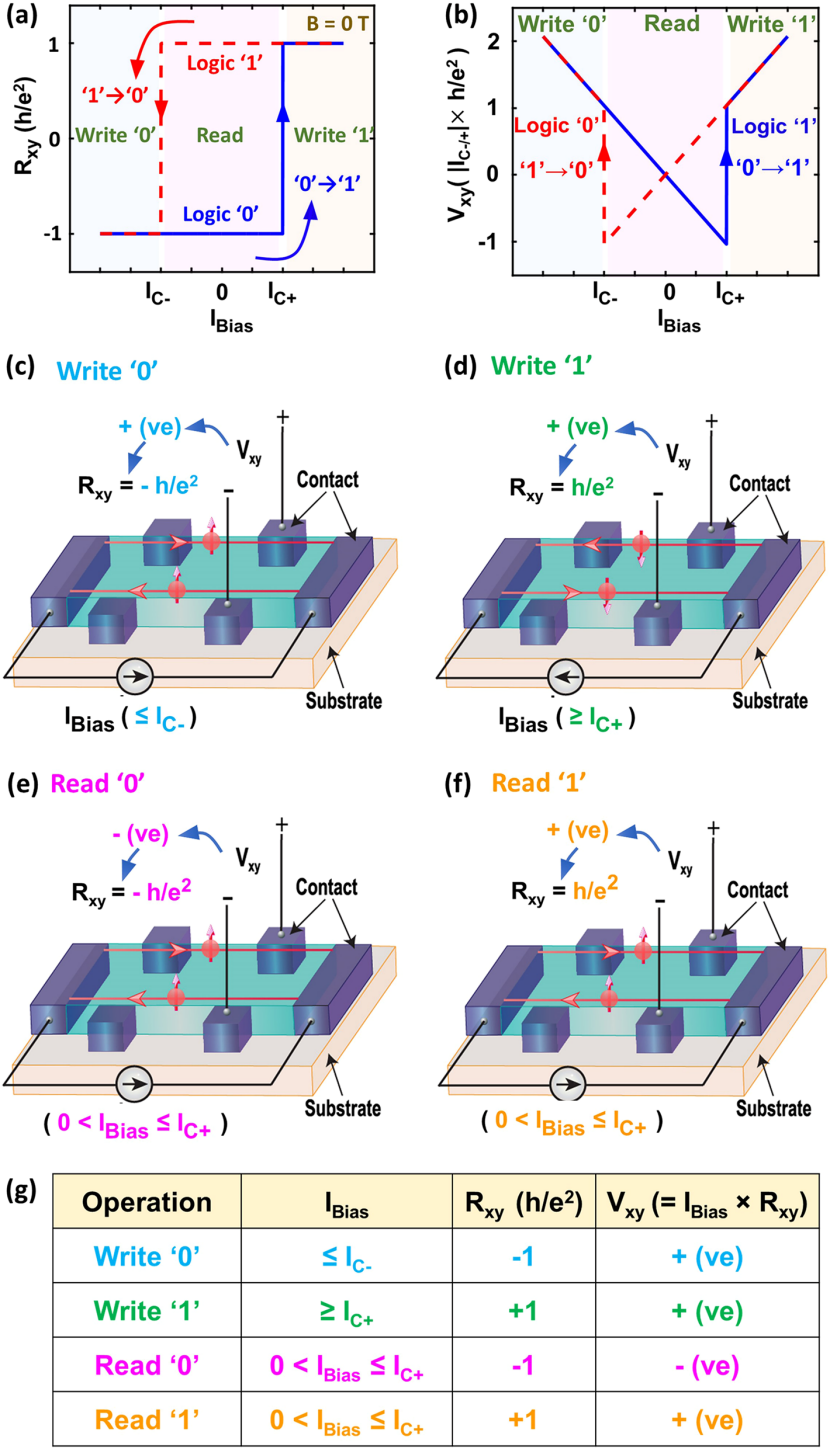
Illustration of the quantum anomalous Hall effect based memory operation. (**a**) *R*_*xy*_ vs. *I*_*Bias*_ at zero external magnetic field. Hall resistance values of *− h/e*^*2*^ and + *h/*e^2^ are defined as logic ‘0’ and logic ‘1’ respectively. Three regions: (i) *I*_Bias_ ≤ *I*_*C-*_, (ii) *I*_Bias_ ≥ *I*_*C*+_, and (iii) *I*_*C−*_ < *I*_Bias_ < *I*_*C*+_ are marked as write ‘0’, write ‘1’ and read region, respectively. We use this division to choose the required *I*_Bias_ for different memory operations. (**b**) Hall voltage, *V*_*xy*_ plotted as a function of *I*_Bias_. (**c**), (**d**), (**e**), & (**f**) The procedures of write ‘0’, write ‘1’, read ‘0’, and read ‘1’ operations, respectively, along with the state of electron spins (red arrows). (**g**) Summary of the key idea for the QAHE based memory. The table enlists the required ranges of *I*_Bias_ corresponding to all memory operations, along with the corresponding Hall resistance and Hall voltage levels.

Figure 2 captures the highlight of our idea to leverage the quantized Hall resistance of a QAH insulator as a non-volatile memory cell along with the write and read operations. We define the quantized Hall resistances, *−* *h/e*^*2*^ and + *h/e*^*2*^*,* as logic ‘0’ and logic ‘1’ respectively. Based on this definition, *I*_Bias_ can be divided into three regions (Fig. 2a)—(i) *I*_Bias_ ≤ *I*_*C-*_: write ‘0’ region, (ii) *I*_Bias_ ≥ *I*_*C*+_: write ‘1’ region, and (iii) *I*_*C-*_ < *I*_Bias_ < *I*_*C*+_: read region. Figure 2b shows the bias current dependence of the Hall voltage, *V*_*xy*_ (= *I*_Bias_ × *R*_*xy*_). Importantly, as seen in Fig. 2b, write operations of both logic ‘0’ and ‘1’ entail positive *V*_*xy*_ because *I*_Bias_ and R_xy_ are either both positive (when writing logic ‘1’; Fig. 2c), or both negative (when writing logic ‘0’; Fig. 2d). In sharp contrast, read operations of logic ‘0’ and ‘1’ manifest opposite sign of *V*_*xy*_. For example, if we use a positive bias current (*0* < *I*_Bias_ < *I*_*C*+_*)*, logic ‘1’ and logic ‘0’ correspond to positive and negative *V*_*xy*_, respectively. The clear difference in the sign of the Hall voltage for logic ‘0’ and ‘1’ makes the sensing of the memory states simple and straightforward. Figure 2g summarizes the key idea of a non-volatile memory utilizing the quantization of the Hall resistance in a QAH insulator, listing *I*_Bias_ for write and read operations, as well as the state of the Hall resistance and Hall voltage for the two memory states.

## Operation of a QAHE based memory

**Figure 3:**
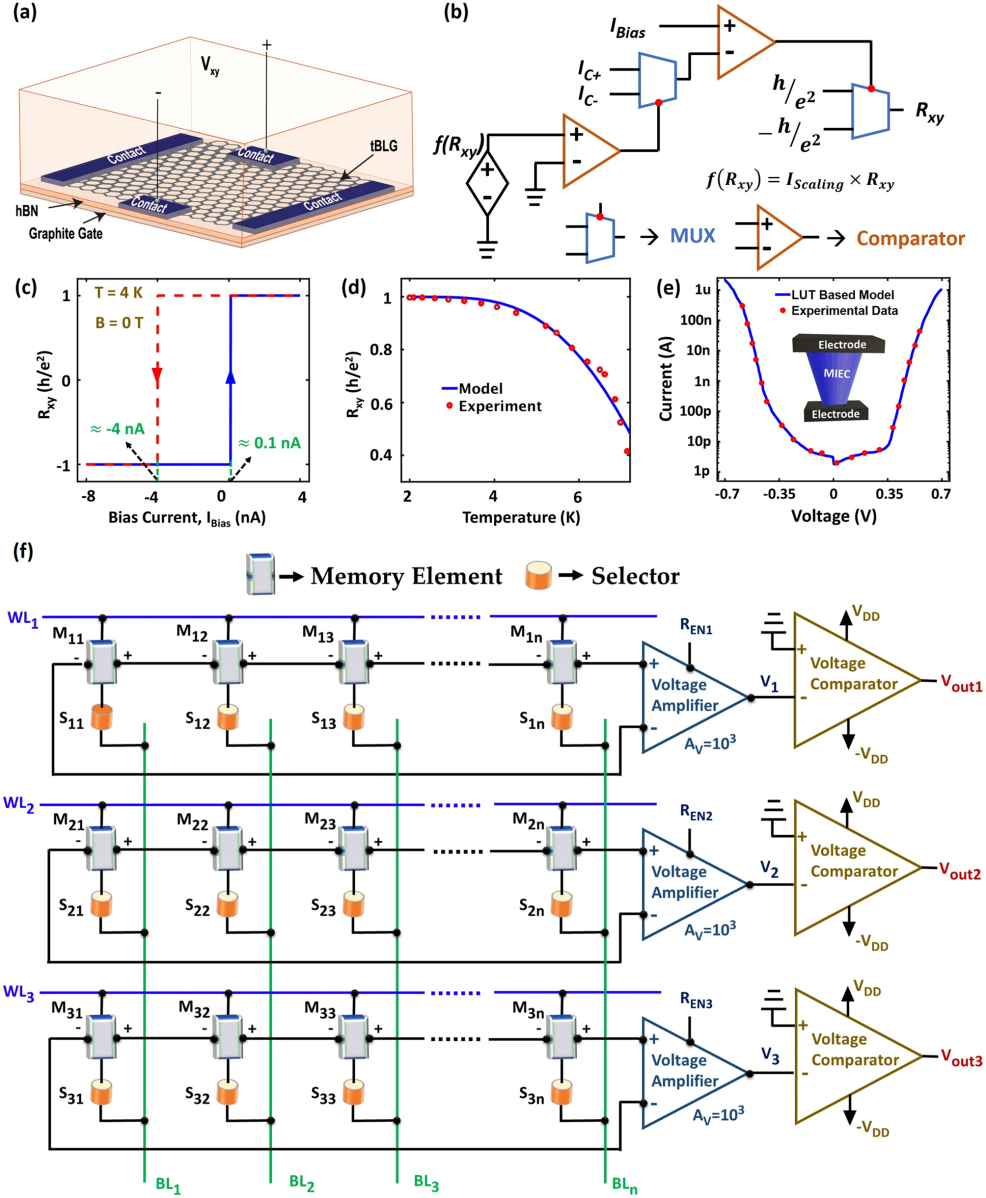
Design of the 3D cross-point memory array structure using a tBLG moiré heterostructure as a memory element and mixed-ionic-electronic-conduction based access device as selector. (**a**) Schematic of a tBLG moiré heterostructure where tBLG is encapsulated between flakes of hBN and a flake of few-layer graphite is used as gate. (**b**) Behavioral representation of our phenomenological model of the observed QAHE in the tBLG moiré heterostructure. (**c**) *R*_*xy*_ data*,* plotted as a function of *I*_Bias_ at T = 4 K and B = 0 T, exhibit current driven hysteretic switching. (**d**) Temperature dependence of *R*_*xy*_ with an experimental matching. (**e**) *I–V* characteristics obtained from a look-up table (LUT) based model of the mixed-ionic-electronic-conduction (MIEC) based selector device. (**f**) Illustration of the overall memory array structure, custom-designed for the QAHE based memory devices.

It is worthwhile to analyze the operation of the proposed QAHE based non-volatile memory in a 3D cross-point memory array. Figure 3a schematically shows our memory element: a tBLG moiré heterostructure where tBLG (with interlayer twisted angle of 1.1$^\circ$) is encapsulated between flakes of hBN^14^. We have developed a Verilog A based phenomenological model for the QAHE in tBLG moiré heterostructure to use in our analysis of the heterostructure as a memory cell. The model can also be calibrated for other QAH insulators using the values of bias currents (*I*_*C-*_ and *I*_*C*+_) and the Chern number ($\nu$). A behavioral representation of our model is shown in Fig. 3b. Figure 3c shows *R*_*xy*_ as a function of *I*_Bias_ at zero external magnetic field and at T = 4 K which is obtained from the model shown in Fig. 3b. As seen from Fig. 3c, we can deduce the values of *I*_*C-*_ and *I*_*C*+_ for the tBLG heterostructure (approximately − 4 nA and 100 pA, respectively) which are crucial for the memory operation. In our phenomenological model, we also account for the temperature dependence of Hall resistance using the following Eq. ^1^:

1 $$R_{xy} = \frac{h}{{e^{2} }} - R_{1} e^{{ - \frac{{\Delta }}{T}}} ,$$

where *R*_*1*_ is a fitting constant, $\Delta$ (= 26 ± 4 K) is the energy required to create and separate an excitation of particle-antiparticle of the QAH state and T is the temperature. Figure 3d, which shows the temperature dependence of the Hall resistance obtained from our model, agrees reasonably with the measured values reported in Ref.^1^.

### Design of a cross-point array

To evaluate the practicality of a memory device, it is crucial to consider an array level scenario. The unique properties of our QAHE based memory device necessitate a custom designed memory array. To ensure the highest storage density, we adopt the cross-point memory architecture^26^ with slight modification in the inter-cell connection pattern. A major component of the cross-point array is a two-terminal selector device^27^ that allows access to a specific cell for read/write operation and suppresses current flow through the other cells. The selector devices are connected in series with the memory elements in every cell. A multitude of selector devices exist^28–33^ with diverse selectivity (ON/OFF ratio) and switching thresholds. For our QAHE based memory, the Cu-containing mixed-ionic-electronic-conduction (MIEC) material will be a perfect selector, due to its high selectivity (~ 10^6^), and ultra-low leakage (< 10 pA)^34–36^. We have developed a look-up-table based phenomenological model (in Verilog-A) for this selector and calibrated the model (Fig. 3e) with the measured current–voltage (*I–V*) characteristics reported in Refs.^34–36^.

Figure 3f shows the schematic view of our proposed array architecture. Every memory cell (QAHE device + selector) is sandwiched between orthogonally running metal lines, namely word line (WL) and bit line (BL). The WLs and the BLs are shared along the rows and columns of the array, respectively. The difference between the DC voltages applied on the WL and BL of a particular a memory cell essentially dictates the effective voltage across the cell (*V*_*Cell*_). Note, the substrate terminal of every cell is biased with a constant voltage to maintain an appropriate electron density [not illustrated in the figures for simplicity]. Conveniently, we need only one control input, *V*_*Cell*_, to read from or write into the QAHE devices. We utilize three different levels of *V*_*Cell*_ (with appropriate polarity) to write logic ‘1/0’ and to read the stored data.

Our architecture entails accessing one cell per row at a time. The Hall-voltage terminals of the neighboring cells in the same row are electrically connected in series with each other (Fig. 3f). Importantly, the resultant voltage across the end terminals of the series connected cells in a row (${V}_{hr}$) bears the signature of the stored data in the accessed memory cell, along with some residual contributions from the other cells in the same row. For example, consider that every row holds *n* cells, and the *k*th cell of the *i*th row is being accessed. The resultant terminal voltage for this row can be expressed as:

2 $${V}_{hr-i}=\sum_{j=1}^{n}{V}_{xy-ij}-{2n\times V}_{loss }={I}_{\mathrm{Bias}-ik}\times {R}_{xy-ik}+\sum_{j=1, j\ne k}^{n}{I}_{\mathrm{Bias}-ij}\times {R}_{xy-ij}-{2n\times V}_{loss }$$

here ${V}_{loss}$ denotes the average loss component per contact, indicating the inherent non-ideality. The specialized biasing scheme of the memory array (discussed later) ensures that the losses and residual components are negligible (quantified in the Sect. 3 of the Supplementary Materials) compared with the Hall voltage of the accessed cell. As a result, the polarity of *V*_*hr*_ is dictated by the *R*_*xy*_ of the accessed cell. For the tBLG moiré heterostructure based QAHE device, we need to amplify the *V*_*hr*_ from a few tens of microvolts to tens of millivolts using cryogenic amplifiers. A suitable candidate for such an amplifier can be one of the cryogenic low-noise amplifiers reported in Refs.^37–39^. After the amplification, the millivolt level Hall voltage is used to determine the memory state of the accessed cell. Recall, during read operation, logic ‘0’ and ‘1’ memory states correspond to opposite polarity of Hall voltage (Fig. 2b). Therefore, we feed the amplified *V*_*hr*_ to a cryogenic voltage comparator^40^ to sense the memory state of the accessed cell.

### Read/write operations

**Figure 4:**
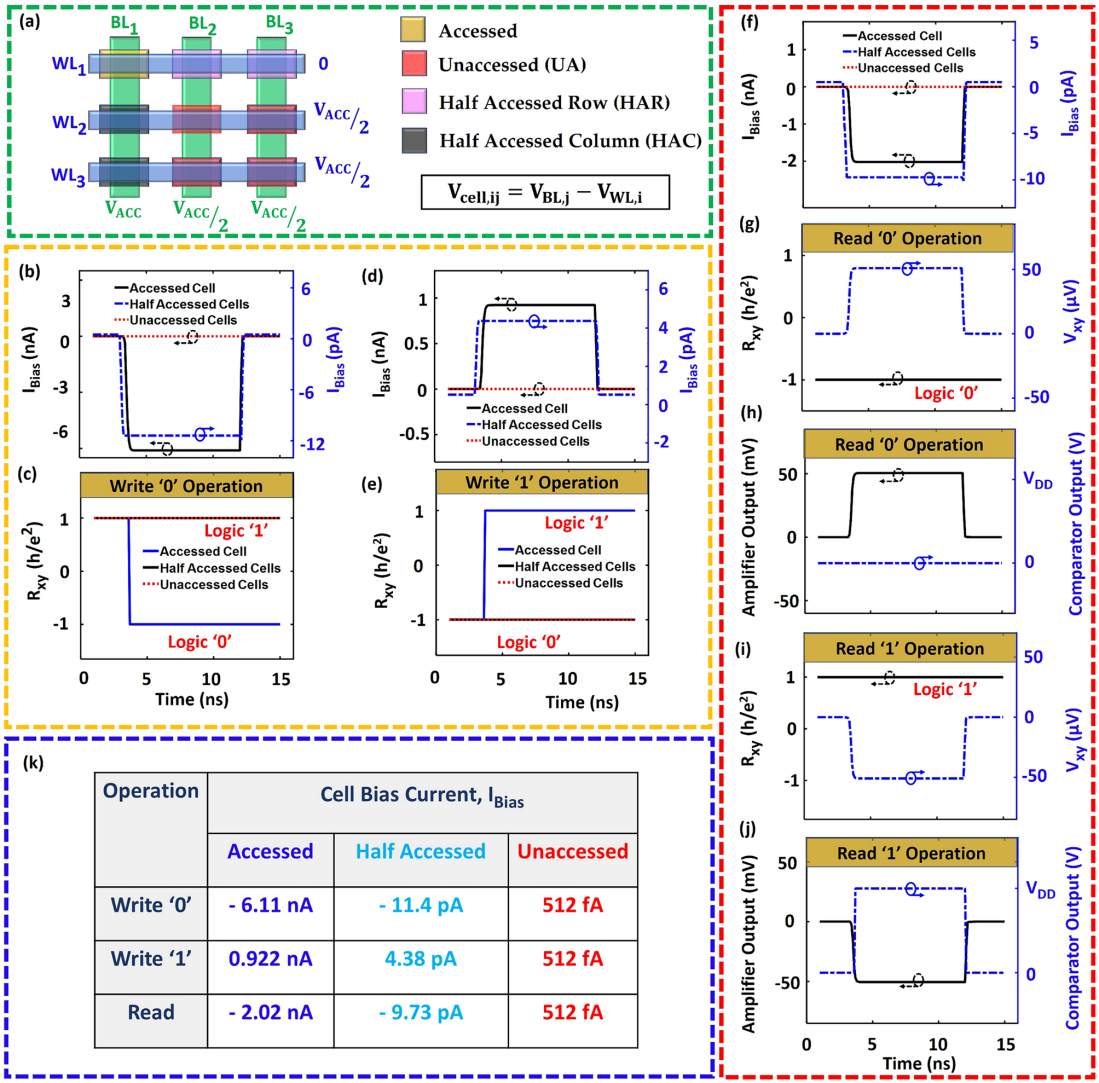
Compilation of the simulation results for the memory array. (**a**) Arrangement and types of memory cells in a conventional cross-point memory array under the *V/2* biasing scheme. *Write ‘0’ operation*: (**b**) Cell Bias currents (*I*_Bias_) through different memory cells generated by applying appropriate *V*_*ACC*_ **(c)** The time dynamics of the Hall resistance (*R*_*xy*_) illustrating state transition only in the accessed cell. *Write ‘1’ operation*: (**d**) *I*_Bias_ with different magnitude and opposite polarity (compared with the write ‘0’ case) flowing through different memory cells (**e**) Corresponding transition in *R*_*xy*_*. Read operation*: (**f**) The *I*_Bias_ is of the same polarity as the write ‘o’ operation, but has ~ 3X lower magnitude. The peripheral circuits (amplifier and comparator) are enabled only during this period. The comparator output is always initialized at zero volage. (**g**) If the memory cell holds binary ‘0’, the Hall voltage (*V*_*xy*_) exhibits a positive polarity during the read operation. *R*_*xy*_ remains unchanged throughout the process. (**h**) The amplified *V*_*xy*_ is fed into a comparator which produces a digital output. While reading ‘0’, the comparator output remains unchanged at its initial value (0 V). (**i**) Read ‘1’ operation produces the opposite polarity in *V*_*xy*_, and *R*_*xy*_ remains unchanged. (**j**) The amplified *V*_*xy*_ triggers a 0→V_DD_ transition in the comparator output, which remains stable throughout the read operation.

In Fig. 4, we present the simulated memory operations (read/write) in our proposed QAHE based cross-point array. Figure 4a illustrates four types of memory cells in a block of cross-point array. We utilize a standard biasing scheme for cross-pint arrays, commonly known as the *V/2* biasing^33^. Different levels of access voltage (*V*_*ACC*_) are applied to read from or write into a specific cell. The row (column) that holds the accessed cell is called the half-accessed row (column), because the inactive cells in this row (column) receive half of the access voltage (*V*_*ACC*_*/2*). All the other unaccessed cells ideally have zero voltage across them. Without any loss of generality, we assume M_11_ (Fig. 3f) to be the accessed cell and examine its read/write dynamics. Figure 4b shows the cell current levels through the accessed, half-accessed, and unaccessed cells during 1→0 memory write operation. Only the accessed cell exhibits a transition (+ h/e^2^ → − h/e^2^) in the Hall resistance (*R*_*xy*_) (Fig. 4c), indicating a successful write operation in the accessed cell without disturbing the other cells. Figure 4d,e show similar time dynamics for the 0→1 write operation. Note, the cell current level for the 0→1 operation is of the opposite polarity compared to the 1→0 operation (Fig. 4d). The repeated write operations (0→0 and 1→1) have also been tested and are presented in supplementary Fig. S2.

Next, we examine the read dynamics (Fig. 4f–j) of the proposed memory cell. The current flow direction for the read operation is the same as the 1→0 write operation (Fig. 4f). However, the magnitude of the read current is about one-third of the critical current of transition (I_C-_), which alleviates the possibility of accidental 1→0 data flip. In addition, the chosen polarity of the read current nullifies the possibility of accidental 0→1 transition. The read current through the QAHE device generates a Hall voltage (*Vxy*), whose polarity is dictated by the previously set Hall resistance ((+ h/e^2^ or − h/e^2^). In our design, positive/negative polarity of *Vxy* corresponds to logic 0/1, respectively (Fig. 4g,i). It is worth noting, the read operation utilizes different pair of terminals than the write operation, allowing independent optimization of read/write peripheral circuits. Furthermore, the amplifier-comparator pairs are enabled only during the read operation to minimize peripheral power demand. The comparator in our design also provides digital outputs corresponding to the stored data in the accessed cell (Fig. 4h,j). Note, the resistance of the MIEC exponential selector drastically increases for lower voltage range (< 0.35 V), as shown in supplementary Fig. S5. The array biasing scheme is designed to enforce high resistance mode of the selector in every memory cell but the accessed cell. This design technique ensures that the half-accessed and unaccessed cells encounter orders of magnitude less current compared with the accessed cell. The MIEC selector takes an overwhelming share of any voltage applied across the cell, averting the chances of a breakdown in the QAHE device. The bias currents that flow through the cells during different memory operations are shown in Fig. 4k which clearly demonstrate that the memory states of the half-accessed and unaccessed cells will not be disturbed during write or read operation in the accessed cell. Thus, our architecture lays out a device-to-array design pathway for the QAHE based unique memory devices.

## Outlook and discussion

Our cryogenic memory framework is transferable to the material structures that exhibit current-controlled switching of QAH states (*e.g.,* tri-layer graphene^24,25^). Some of these material structures will necessitate an initialization step to first create a magnetic moment in the system. The additional analog components required for this purpose will be parts of the array peripherals. The underlying transition mechanisms of the QAHE devices may determine their endurance and reliable life span.

We close by discussing the broad impact of our work. This manuscript makes the first mark to construct a working electronic device using topological properties of materials. Here we leverage QAHE, that does not require an external magnetic field, and design an elegant memory device that can be built with topological quantum materials. The proposed non-volatile memory architecture is a strong candidate for the cryogenic memory system, thanks to its ultra-low temperature compatibility. Note, the cryogenic memory block is a crucial component of quantum computing systems based on superconducting qubits^41^. Our proposed QAHE-based memory device offers a significant reduction in the cell area and 1000 times reduction in the cell read/write power compared with the state-of-the-art cryogenic memory devices^42–49^ (see supplementary Table S1 for detailed comparison). Our proposed memory device is a potential game-changer for scalable quantum computing systems^41^ and space cryogenics^50^.

## Supplementary Information

Supplementary Information

## Data Availability

The data that support the plots within this paper and other findings of this study are available from the corresponding author upon reasonable request.
