# Supplementary material for: A non-volatile cryogenic random-access memory based on the quantum anomalous Hall effect
Source: Sci Rep. 2021 Apr 12;11:7892. doi: 10.1038/s41598-021-87056-7 (PMC8042021; doi:10.1038/s41598-021-87056-7)
Supplement: Supplementary file 1 — Supplementary Information [file 41598_2021_87056_MOESM1_ESM.pdf]

Supplementary Materials: A Non-Volatile Cryogenic Random-Access Memory Based on Quantum Anomalous Hall Effect

Shamiul Alam[1†], Md Shafayat Hossain[2†], and Ahmedullah Aziz[1*]

[1]Dept. of Electrical Eng. & Computer Sci., University of Tennessee, Knoxville, TN, 37996, USA

[2]Dept. of Electrical Engineering, Princeton University, Princeton, NJ, 08544, USA

† These authors have contributed equally

1. Phenomenological Model of Quantum Anomalous Hall States in tBLG Moiré Heterostructure

We have developed a Verilog-A based phenomenological model for the recently observed intrinsic quantum anomalous Hall effect (QAHE) in tBLG moiré heterostructure, and demonstrate the operation of the proposed QAHE-based memory device [1]. In the main text, we have shown the DC bias current dependence of Hall resistance obtained from our model. Here, in the supplementary Fig. S1, we highlight a few additional device characteristics obtained from the model. In Figs. S1(a) & (b), we show the AC bias current dependence of Hall resistance [Fig. S1(b)] at 4 K temperature where a 40 $nA_{p\text{-}p}$ AC bias current [Fig. S1(a)] has been applied. Another feature of our model is the incorporation of the temperature dependence of the Hall resistance. To demonstrate the temperature dependence of the Hall resistance, in Fig. S1(c) we show the DC bias current dependence of the Hall resistance at different temperatures obtained from our model.

2. Feasibility of the Approach Used for Write and Read Operation

In the main text, we have presented the read and write operation for a cell in a 3D cross-point memory array. During the read operation from a cell, the Hall voltage terminals of the neighboring cells in the same row are electrically connected in series. Then, we use the algebraic sum of the Hall voltages of the cells in a row (V_{hr}) to sense the memory state stored in a particular cell of that row. Note that positive/negative Hall voltage corresponds to logic '0'/'1' for the read operation. Notably, during the memory operations, it is important that *(i)* the sign of V_{hr} remains same as that of the Hall voltage of the accessed cell, and *(ii)* the current that flows through the half-accessed and unaccessed cells during read and write operations do not affect the memory states stored in those cells. Figures S2(d) and S2(e) depict the repeated write operations (write 0 → 0 and 1 → 1) of the accessed cell. It is also clear from these figures that the Hall resistance of the other cells remain undisturbed. We also show the Hall voltages across the half-accessed and unaccessed cells during write '0' [Fig. S2(f)], write '1' [Fig. S2(g)], and read operation when logic '1' [Fig. S2(h)] and logic '0' [Fig. S2(i)] are stored in the half-accessed and unaccessed cells. As seen in these figures, the Hall voltages across the half-accessed row (HAR) cells are in nano-volt range during all the operations. Moreover, in Fig. 4(k) of the main text, we show the cell bias currents through all the cells during different memory operations. The choice of the bias current value further ensures that the stored memory sates in the HAR, half-accessed column (HAC) and unaccessed cells are not disturbed during read and write operations in a particular accessed cell.

3. Case for Non-ideality of the Contacts

Non-ideal contacts are a cause for concern in most designs, including ours. In our cross-point array (Fig. 3), we establish a series connection between the QAHE cells in the same row to read just 'one' cell at a time. Then the two end nodes of the series connected cells (for 1st row, the '+' contact of the M1n cell and the '-' contact of M11 cell) are fed into a voltage amplifier. In our case, op-amp based designs are the best choices. Fig. S3 shows the connection of the series connected cells (4×4 array) with the voltage amplifier for the first row. Here, an op-amp based non-inverting amplifier has been used. Now, according to the

CMOS architecture of an op-amp [Fig. S4(a)] reported in 3, the two contacts ('+' contact of the last cell and '-' contact of the first cell of a row) of the series connected cells are connected to two gate terminals of two MOSFETs [M1, M2 in Fig. S4(a)]. Ideally no current flows through the gate terminal of a MOSFET. Practically, however, a very small current flows (gate leakage components) and the resistance between the gate and body terminal (typically connected to the ground) of a MOSFET is very high (typically in mega-ohm range). Therefore, a very small (quantified later) amount of current will flow through the cells.

To further clarify, here we show a calculation of current flow through the cells of a 4×4 array in Fig. S4(b). Let us consider the worst-case scenario, i.e., when maximum current flows through the cells. RGB1 and RGB2 are the gate to body resistances (~ 10 MΩ, estimated for a typical gate stack) of two MOSFETs where the sum of the cell voltages is connected. calculation shows that, such a connection will give rise to ~2.64 pA of current through the series connected memory chain.

Now, let us bring in contact resistance in consideration. Considering the worst-case scenario where the contact resistance is around 100 Ω, the extra loss at the contacts will still be smaller than a nano-volt. As shown in Fig R1 (b), the accessed cell will create more than 5000× voltage than this loss component. Considering 2 contacts per cell, we can accommodate thousands of cells per row, before being worried about the contact losses. Therefore, for all practical purposes, we can ignore the loss term in Eq. 2 (Main text).

4. Comparison with the State-of-the-art Cryogenic Memories:

Finding a low-power-consuming, high-capacity cryogenic memory is one of the major challenges in implementing a scalable quantum computer [2]. A number of designs based on Josephson junctions and CMOS architecture have been proposed. However, they have critical bottlenecks. While the Josephson junction-based memories are fast and energy efficient, they suffer from low capacity [3,4]. Conversely, CMOS memories lack speed and energy efficiency, albeit they have high capacity. To address this issue, one may think of hybrid memories utilizing the advantages of both the technology [5–8]. Intriguingly, this approach still poses a challenge of finding suitable interface circuits. Our proposed QAHE based memory, on the other hand, shows a strong potential as a cryogenic memory platform in quantum computers and cryogenic electronic systems thanks to its very small area and very low power operations. Moreover, cross-point array structure that we have constructed for this memory bestows the advantage of the high-density random-access memory. In Table S1, we summarize the comparison between our proposed memory and the state-of-the-art cryogenic memories in terms of area and power consumption. Our proposed QAHE based non-volatile memory offers a significant amount of reduction in the cell area and a 1000 times reduction in the cell read/write power compared to the state-of-the-art cryogenic memory devices.

Table S1: Comparison with the state-of-the-art Cryogenic Memories

Cryogenic Memories	Cell Area	Power Consumption
Superconducting Memory Chip [9]	$50 \times 46\ \mu m^2$	1.62 mW for 2Kb RAM<br>[Approximated 0.79 µW per cell]
VT RAM [10]	$55 \times 55\ \mu m^2$	6.7 mW for 4 Kb RAM<br>[Approximated 1.64 µW per cell]
SFQ CRAM [11]	$40 \times 45\ \mu m^2$	2.4 mW for 16 Kb RAM<br>[Approximated 0.15 µW per cell]
High frequency RAM [4]	$55 \times 55\ \mu m^2$	Not Reported
Hybrid Josephson-CMOS Memory [6,7]	$6.5 \times 7.5\ \mu m^2$	0.7 mW for reading and 1.4 mW for writing for 64 Kb RAM<br>[Approximated 0.01 µW for reading and 0.02 µW for writing per cell]
Small Array of Josephson Junctions [12,13]	$5 \times 5\ mm^2$<br>(Chip Size)	~ 5 nW (maximum) for reading and writing per cell (predicted)
QAHE based Proposed Memory (This work)	$5 \times 2\ \mu m^2$	0.96 pW for writing and 0.1 pW for reading per cell (simulated)

5. Non-linear Resistance of the MIEC Material based Selector:

The Cu-containing mixed-ionic-electronic-conduction (MIEC) material based selector shows extremely non-linear resistance response (Fig. S5). These selectors present very high resistance (~ GΩ) within a lower voltage range ($|V| < 350$ mV) and lower resistance (~ MΩ) with higher terminal voltage. This difference leads to the orders of magnitude difference between the accessed and half-accessed cells, as shown in Fig. 4(k) of the main text.

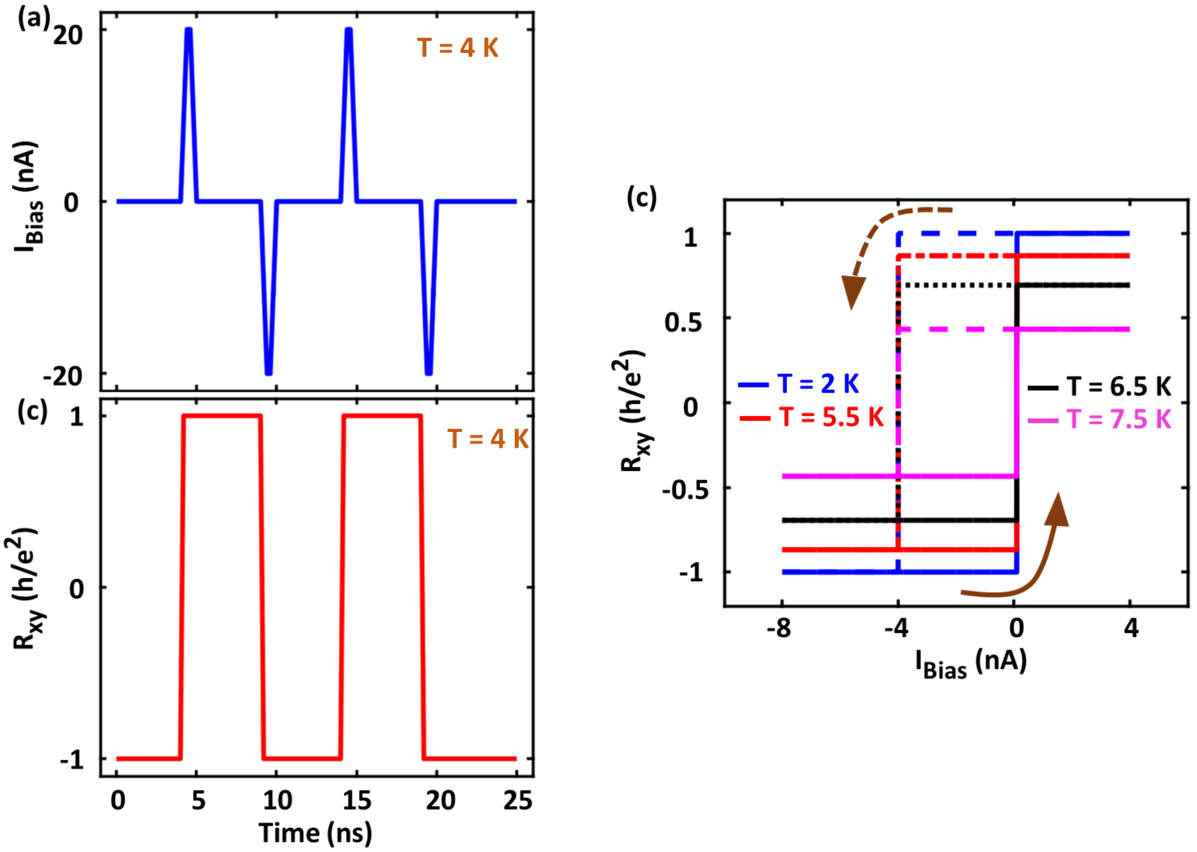

Fig. S1: Time dynamics of the bias current and the Hall resistance and the temperature dependence obtained from our developed phenomenological model for the studied QAHE device. Time dynamics of **(a)** the bias current, I_{Bias} and **(b)** the resulting Hall resistance, R_{xy} in tBLG moiré heterostructure at 4 K temperature. **(c)** Hall resistance, R_{xy} as a function of the bias current, I_{Bias} at different temperatures obtained from the phenomenological model.

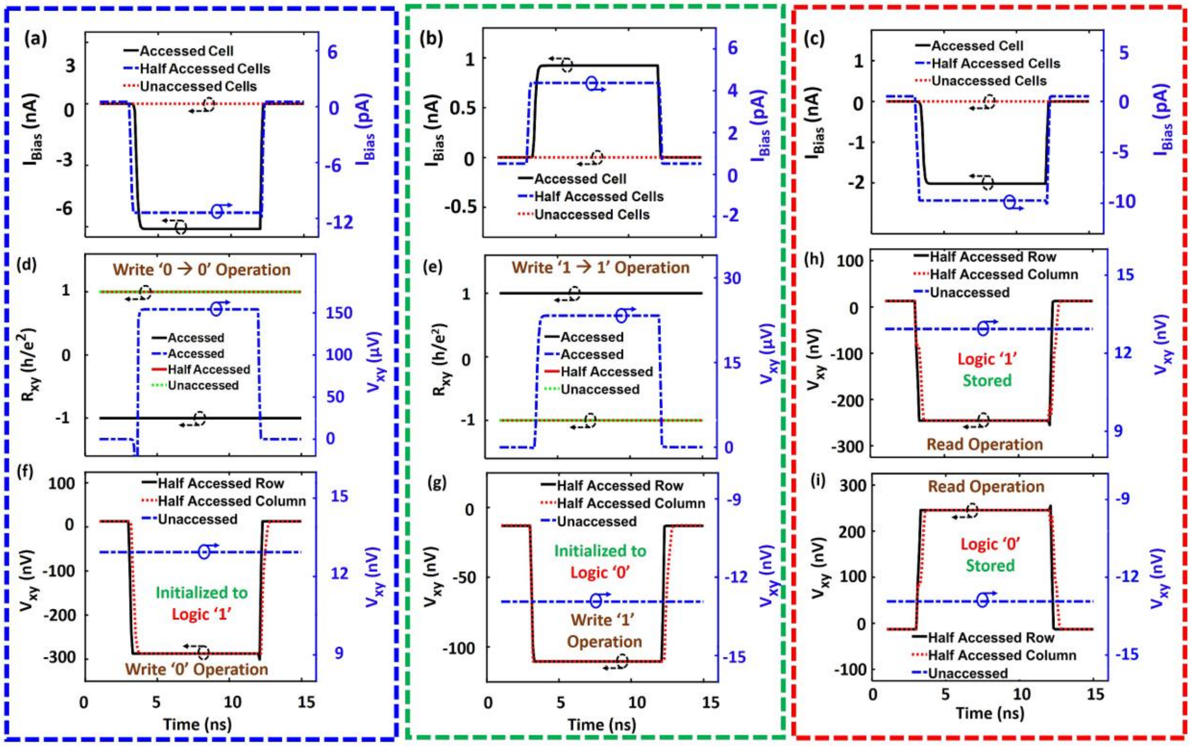

Fig. S2: Additional details for the read/write operations. Cell Bias currents (I_{Bias}) corresponding to the choice of specific values of V_{ACC} during **(a)** write 0', **(b)** write '1', and **(c)** read operations. Time dynamics of the Hall resistance (R_{xy}) and Hall voltage (V_{xy}) across the accessed cell during the '0 → 0' write operation. To examine the possibility of accidental write operations occurring in other cells, we consider that all other cells are storing logic '1' ($R_{xy} = h/e^2$). Clearly, the R_{xy} remains stable not only for the accessed cell, but also for all other cells, as expected. (e) Similar argument applies to the '1→1' write operation in the accessed cell. The V_{xy} levels for the half-accessed and unaccessed cells are limited to sub-microvolts for (f) write '0' and (g) write '1' operations. Although V_{xy} do not play any role in the memory write mechanism, these residual voltage levels lead to mild leakage power loss. Finally, we present the residual V_{xy} levels that appear across the read terminals of half-accessed and unaccessed cells during the (h) read '0' and (i) read '1' operations. Such ultra-low V_{xy} levels ensure low leakage power throughout the array.

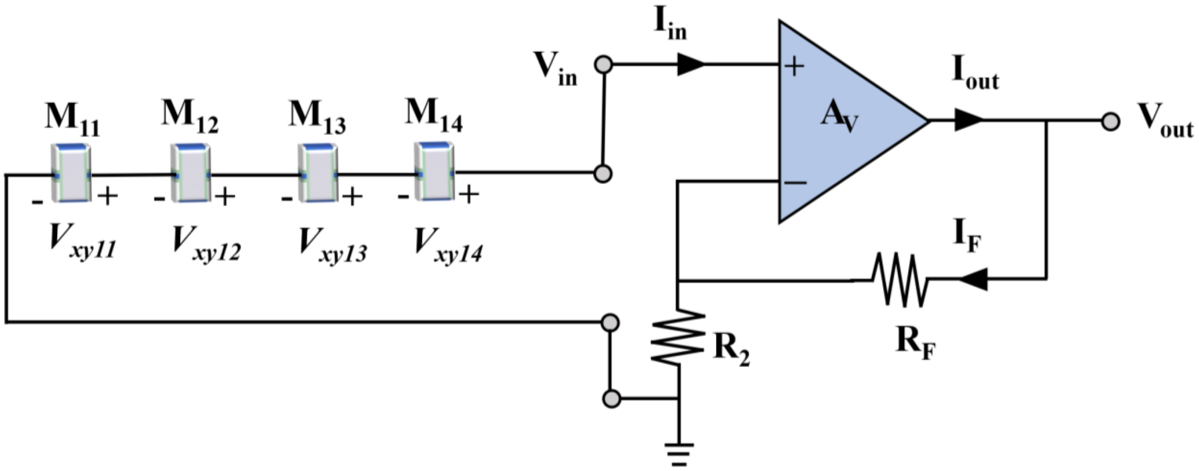

Fig. S3: Connection of the Hall voltages of the series connected cells of the first row of a 4X4 array with the voltage amplifier circuit.

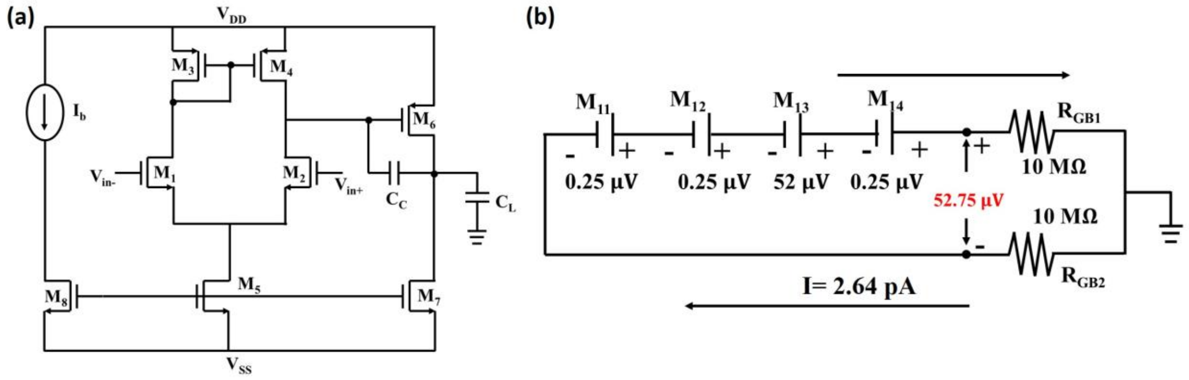

Fig. S4: (a) CMOS architecture of an op-amp, and **(b)** Calculation of current flow through the cells of the first row of a 4X4 array.

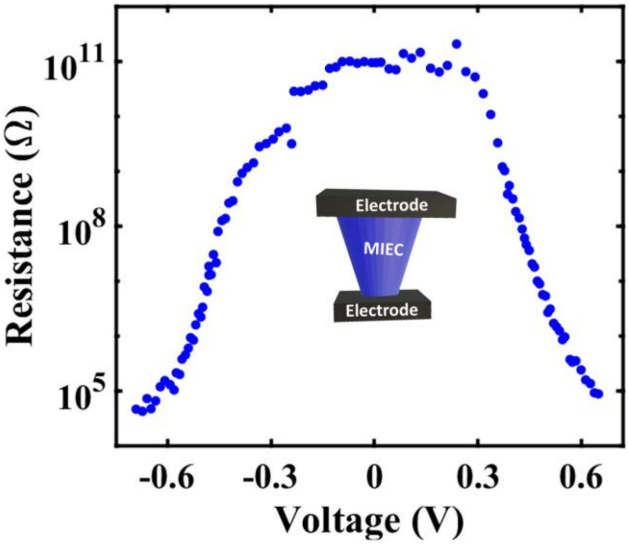

Fig. S5: Non-linear resistance response of the MIEC material-based selector devices.

References:

1. Serlin, M., Tschirhart, C.L., Polshyn, H., Zhang, Y., Zhu, J., Watanabe, K., Taniguchi, T., Balents, L., Young, A.F., Intrinsic quantized anomalous Hall effect in a moiré heterostructure. *Science* **367**, 900–903. doi:10.1126/science.aay5533 (2020).
2. Tolpygo, S. K. Superconductor digital electronics: Scalability and energy efficiency issues. *Low Temperature Physics*, **42**, 361-379, doi:10.1063/1.4948618 (2016).
3. Polonsky, S.V., Kirichenko, A.F., Semenov, V.K. and Likharev, K.K. Rapid Single Flux Quantum Random Access Memory. *IEEE Transactions on Applied Superconductivity*, **5**, 3000-3005, doi:10.1109/77.403223 (1995).
4. Nagasawa, S., Numata, H., Hashimoto, Y. and Tahara, S. High-frequency clock operation of josephson 256-word x 16-bit rams. *IEEE transactions on applied superconductivity*, **9**, 3708-3713, doi:10.1109/77.783834 (1999).
5. Ghoshal, U., Kroger, H. and Van Duzer, T. Superconductor-Semiconductor Memories. *IEEE Transactions on Applied Superconductivity*, **3**, 2315-2318, doi:10.1109/77.233542 (1993).
6. Liu, Q., Fujiwara, K., Meng, X., Whiteley, S. R., Van Duzer, T., Yoshikawa, N., Thakahashi, Y., Hikida, T. & Kawai, N. Latency and power measurements on a 64-kb hybrid Josephson-CMOS memory. *IEEE Transactions on Applied Superconductivity*, **17**, 526-529, doi:10.1109/TASC.2007.898698 (2007).
7. Feng, Y. J., Meng, X., Whiteley, S. R., Van Duzer, T., Fujiwara, K., Miyakawa, H. & Yoshikawa, N. Josephson-CMOS hybrid memory with ultra-high-speed interface circuit. *IEEE Transactions on Applied Superconductivity*, **13**, 467-470, doi:10.1109/TASC.2003.813902 (2003).
8. Yau, J.B. and Gibson, G.W. Hybrid cryogenic memory cells for superconducting computing applications. *IEEE International Conference on Rebooting Computing, ICRC*, **2017**, 1-3, doi:10.1109/ICRC.2017.8123684 (2017).
9. Yuh, P. F. A 2-kbit Superconducting Memory Chip. *IEEE transactions on applied superconductivity*, **3**, 3013–3021, doi: 10.1109/77.257228 (1993).
10. Tahara, S., Ishida, I., Nagasawa, S., Hidaka, M., Tsuge, H. & Wada, Y. 4-Kbit Josephson Nondestructive ReadOut Ram Operated At 580 psec and 6.7 mW. *IEEE transactions on magnetics*, **27**, 2626-2633, doi:10.1109/20.133751 (1991).

11. Kirichenko, A.F., Mukhanov, O.A. and Brock, D.K. A single flux quantum cryogenic random access memory. *Extended Abstract of 7th International Superconductive Electronics Conference*, **1999**, 124-127 (1999).
12. Braiman, Y., Neschke, B., Nair, N., Imam, N. & Glowinski, R. Memory states in small arrays of Josephson junctions. *Physical Review E*, **94**, doi:10.1103/PhysRevE.94.052223 (2016).
13. Nair, N., Jafari-Salim, A., D'Addario, A., Imam, N. & Braiman, Y. Experimental demonstration of a Josephson cryogenic memory cell based on coupled Josephson junction arrays. *Superconductor Science and Technology*, **32**, doi:10.1088/1361-6668/ab416a (2019).
